# Food Allergy: A Rare Cause of Recurrent Intussusception

**DOI:** 10.21699/ajcr.v8i1.513

**Published:** 2017-01-05

**Authors:** Emrah Aydın, Ömer Faruk Beşer

**Affiliations:** 1 Pediatric Surgery Department, Bahcelievler State Hospital, Bagcilar Education and Training Hospital, Istanbul, Turkey; 2 The Center for Fetal, Cellular and Molecular Therapy, Cincinnati Fetal Center, Division of Pediatric General and Thoracic Surgery, Cincinnati Children’s Medical Center, Ohio USA; 3 Pediatric Gastroenterology Department, Okmeydani Education and Training Hospital, Istanbul, Turkey

**Keywords:** Intussusception, Recurrent, Non IgE food allergy

## Abstract

Recurrent intussusception is a management dilemma and have many causes. We report a 22-month old boy who presented multiple times for recurrent intussusception. At diagnostic work-up he was found to be suffering from non-IgE food allergy. The child did not develop further episodes of intussusception after removal of allergenic diet.

## INTRODUCTION

Food allergy is defined as the adverse immunological reaction to a specific food that is reproducible on repeat exposure to the same food.[1] Dietary protein allergy usually develops before 6 months of age. Exposure to these allergens after one year of age is often well tolerated. The allergic response is mostly self-limited, but in some cases it may cause intractable diarrhea and failure to thrive. We present a patient who had three episodes of intussusception in a month and diagnosed as having non-IgE food allergy being probable trigger for intussusception.

## CASE REPORT

A 22-month old boy was referred with colicky abdominal pain and bilious vomiting for a day. He was operated (pneumatic reduction) for intussusception, a week ago. He was otherwise a healthy child. He had tenderness and a palpable mass at right upper quadrant of the abdomen. There was no history of rectal bleeding. Laboratory tests were normal in range including eosinophil count with slightly raised white cell count. There were dilated intestinal loops on abdominal x-ray. Abdominal ultrasonography revealed intussusception. Pneumatic reduction was performed; the patient was discharged on the next day. Ten days after second operation, he again admitted for the same complaints and diagnosed as having ileocolic intussusception. Abdominal CT scan revealed intussusception and enlarged abdominal lymph nodes. Due to multiple recurrence in a short time, abdominal exploration was performed. Multiple lymph nodes, most of which were conglomerated and over 2cm in diameter were found, but no intussusception noted. Appendectomy and lymph node biopsy were performed. He was discharged again in good condition. Histopathologic examination showed lymphoid hyperplasia with eosinophilic infiltration. Two weeks later, he again developed intussusception. Further work-up, with consultation from gastroenterology department, showed eosinophil 3.3% (normal), total IgE 31.53 kU/L (Normal < or =97 kU/L), eosinophil cationic protein 15µg/L (Range 2.3-16 µg/L) and specific IgE for milk, egg, wheat and fish <0,10 kU/L (<0.35 kU/L). He was diagnosed as having non-IgE food allergy per The European Society for Pediatric Gastroenterology Hepatology and Nutrition (ESPGHAN) criteria.[1] Six most common allergens (cow's milk, wheat, egg, shellfish, peanut and fish) were removed from his diet. He became free of any abdominal symptoms for the next month. A provocation test was performed and eliminated allergens were added to his diet. One week later he again developed intussusception which settled on removing allergenic food from his diet. The child is doing fine for the last 10 months on removal of allergenic diet.

## DISCUSSION

Food allergy is characterized by an abnormal immunologic reactivity to food proteins. Gastrointestinal system itself is an immunologic organ. It was once believed that food allergy is mediated by IgE, but nowadays it is shown that non IgE mediated mechanisms can also be involved. There are many cases in the literature related with eosinophilic gastroenteritis accompanied by intestinal obstruction.[2]

Our patient developed three episodes of intussusception in a month. Pneumatic reduction was performed in the first two attacks successfully without any difficulty. Exploration was decided due to suspicion of some lead point. Due to enlarged lymph nodes, we also suspected for any malignancy which was ruled out on histopathology. The histopathological examination revealed increased eosinophils in the specimen. These findings led us think about food allergy. Even though IgE levels and specific immunoglobulin levels were below their limits, elimination of the allergens resolved all symptoms. Also after being asymptomatic for a month on removal of allergenic diet, a provocation tests again developed sonographically proven intussusception with same symptoms. He was diagnosed having as non-IgE food allergy according to ESPGHAN criteria. It seems a diagnosis of exclusion in our patient well supported by provocation test.

Non-IgE mediated food allergy may produce many gastrointestinal tract related symptoms, however, its presentation as intussusception is not reported in children so far.[2] The underlying mechanism is not well known. It is suggested that local production of food specific IgE antibodies might be involved. This local inflammation is supposed to be the “lead point” for intussusception in our case. In conclusion, food allergy should be suspected in cases with recurrent intussusception to prevent unnecessary surgery.

## Footnotes

**Source of Support:** Nil

**Conflict of Interest:** None declared

